# 4*s* Molecular Orbitals and Strongly Correlated 3d States in TiO*_x_* and VO*_x_*

**DOI:** 10.1021/jacs.5c22806

**Published:** 2026-04-17

**Authors:** Daisuke Takegami, Anna Melendez-Sans, Takashi Miyoshino, Ryo Nakamura, Miguel Ferreira-Carvalho, Georg Poelchen, Chun-Fu Chang, Masato Yoshimura, Ku-Ding Tsuei, Haruka Matsumoto, Asuka Yanagida, Ryota Yoshimura, Suguru Yano, Takumi Iwata, Takuro Katsufuji, Atsushi Hariki, Liu Hao Tjeng, Takashi Mizokawa

**Affiliations:** † Department of Applied Physics, 197516 Waseda University, Shinjuku, Tokyo 169-8555, Japan; ‡ 28270 Max Planck Institute for Chemical Physics of Solids, Nöthnitzer Straße 40, Dresden 01187, Germany; § Department of Physics, Tokyo Metropolitan University, Hachioji 192-0397, Japan; ∥ Institute of Physics II, University of Cologne, Zülpicher Str. 77, Cologne D-50937, Germany; ⊥ 57815 National Synchrotron Radiation Research Center, Hsinchu 30076, Taiwan; # Department of Physics, Waseda University, Shinjuku, Tokyo 169-8555, Japan; ∇ Department of Physics and Electronics, Graduate School of Engineering, 12936 Osaka Metropolitan University, 1-1 Gakuen-cho, Nakaku, Sakai, Osaka 599-8531, Japan

## Abstract

We have investigated the electronic structure of the rocksalt TiO_*x*_ and VO_*x*_ systems using polarization-dependent hard X-ray photoelectron spectroscopy. The ability to disentangle the various subshell contributions allowed us to unveil the presence of cation 4*s* molecular orbitals centered around anion vacancies. We observed a close relationship between the presence of these molecular orbitals and the conductive properties of the oxide material, exemplifying the importance of the 4*s* degrees of freedom for low-valent transition metal compounds. The experimentally vanishing intensity at the Fermi level classifies TiO_*x*_ and VO_*x*_ as bad metals and strongly suggests that stoichiometric and defect-free TiO and VO are Mott–Hubbard insulators.

## Graphical abstract

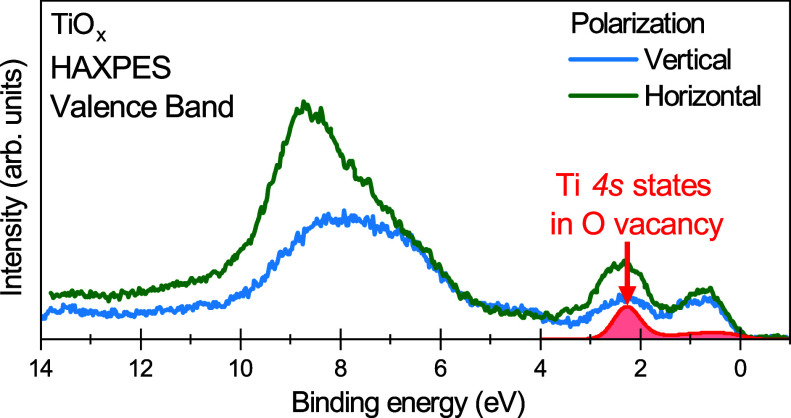

## Introduction

Among the early transition metal oxides (TMOs), TiO_*x*_ and VO_*x*_ based materials have been known for their catalytic activities, for example in the area of oxygen reduction and dehydrogenation processes.^1–5^ However, the fundamental electronic structure of these two oxides is far from clear. This is rooted in the fact that their chemistry is not at all trivial since they seem not to exist as stoichiometric defect-free compounds. In fact, the stability range of both rocksalt TiO_*x*_ and VO_*x*_ is very large with vacancies both on the O and TM sites,^6–15^ highly affecting the conductive properties. Superconductivity even occurs in TiO_*x*_, not only in the bulk material but also in thin films.^16–19^ VO_*x*_ is also peculiar since it is metallic only for *x* ≲ 1, while for higher values of *x* the material displays a more localized behavior.^9,10,12,20,21^

There is thus a long-standing question on the origin of the conductivity in TiO_*x*_ and VO_*x*_. Using the classification scheme of Zaanen–Sawatzky–Allen for TMOs,^22,23^ one could start with the scenario that stoichiometric TiO and VO are Mott–Hubbard insulators in which the TM 3*d* on-site Coulomb energy *U* is larger than both the O-2*p* to TM-3*d* charge-transfer energy and the one-electron bandwidth *W*. The emergence of charge carriers in TiO_*x*_ and VO_*x*_ is then caused by the presence of TM and/or O vacancies. Alternatively, one could envision that the one-electron bandwidth *W* is larger than the on-site Coulomb energy *U* ^16^ so that a *d*-type metal is formed like in NbO,^24^ with the vacancies hampering the conductivity. Numerous theoretical studies have been devoted to this problem,^10,25–31^ but a satisfactory closure is still awaiting. Attempts to study the electronic structure using photoelectron spectroscopy have been challenging due to possible surface and oxidation effects in these particular TiO and VO systems^32,33^ where the presence of vacancies is at the very heart of the problem. In the case of TiO and VO thin films^12,16,19,34,35^ one may have the additional issue that the substrate may alter the stability of the various vacancy states due to strain.

Here we report on our hard X-ray photoelectron spectroscopy (HAXPES) study on single crystalline TiO_*x*_ and VO_*x*_ samples. The bulk-sensitive nature of HAXPES has proven to be highly successful in determining bulk states of many complex TM compounds.^36–38^ Moreover, by making use of the polarization dependence and photoionization cross-section ratios which manifest with better contrast at the higher energies used in HAXPES, we are also able to disentangle the various atomic subshell contributions to the valence band.^38–41^ Contrary to common expectations, we found in TiO_*x*_ and VO_*x*_ a molecular orbital state arising from the TM 4*s* confined around the vacancies, providing an important unexplored degree of freedom beyond the TM *d* and O 2*p* usually considered in describing TMO. We observe a close relation between presence of these vacancy states and the conductive character, i.e., we have identified the source of conductivity in these systems. Finally, we infer from the spectral features and the suppressed intensity at the Fermi level that defect free and stoichiometric TiO and VO should be MH insulators.

## Experimental Section

Single crystal samples of TiO_*x*_ (*x* = 0.93, 1.06, 1.10, 1.28) and VO_*x*_ (*x* = 1.00, 1.15, 1.30) were synthesized as reported in the literature.^15^ HAXPES measurements were performed at the Max-Planck-NSRRC endstation at the Taiwan BL12XU beamline of SPring-8.^40,41^ The photon energy was set to 6.5 keV, with an overall resolution of around 300 meV. The beam is linearly polarized, with one electron energy analyzer located at a direction parallel to the polarization (″horizontal geometry″), and another one perpendicular to the polarization (″vertical geometry″). LDA calculations were performed using FPLO 21 ^42^ using the rocksalt crystal structure with parameters from ref 43, using full relativistic settings.

## Results

### TiO*_x_* Results

Figure 1a shows the valence band spectra of TiO_*x*_ (*x* = 0.93, 1.06, 1.1, 1.28) with two low-energy features α and β at about 0.5 and 2.5 eV, respectively, and two high-energy features γ and δ between 6 and 10 eV binding energy. In titanium oxides, the high-energy features are mainly due to the O 2*p* bands, while closer to the Fermi level they are derived more from the Ti 3*d* bands.^16,33,44,45^ Yet, the presence of the two distinct peaks α and β is very unique and unexpected, and this has been subject of debate in the community.^28,33^ While one may be tempted to assign this two-peak structure as the coherent quasiparticle and incoherent lower-Hubbard bands like in V_2_O_3_,^46,47^ we observe that the intensity near the Fermi level is vanishingly small for all samples as displayed in the inset of Figure 1a. This rather implies a bad metallic behavior for TiO_*x*_ and questions the existence of well-defined quasiparticles. Moreover, the transfer of spectral weight between features α and β as a function of *x* does not follow the trends, qualitatively nor quantitatively, commonly found in doped correlated systems.^48–50^ Instead, features α and β both become substantially weaker with increasing *x*.

**Figure 1:**
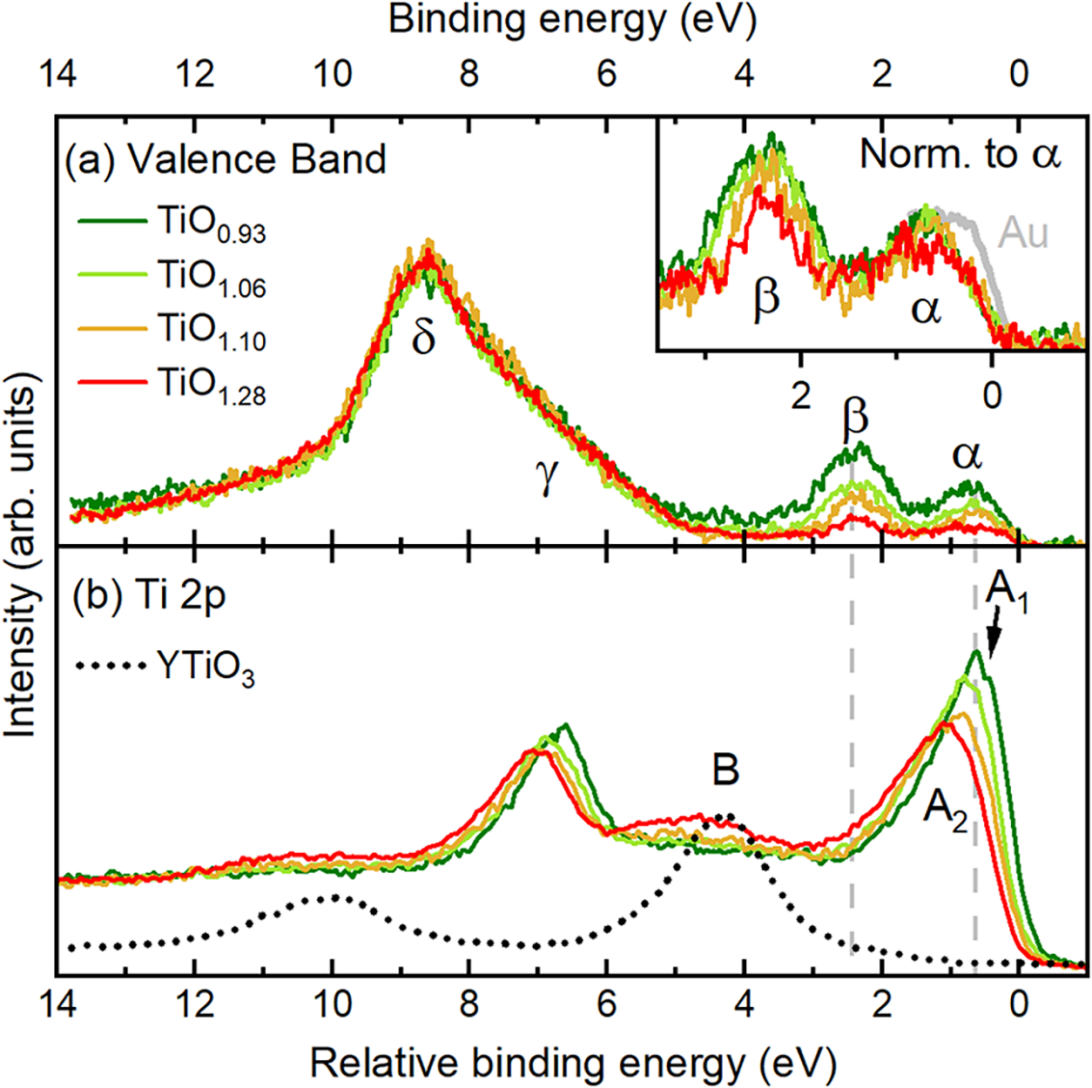
(a) HAXPES valence band spectra of TiO*_x_* (*x* = 0.93, 1.06, 1.1, 1.28), taken with the horizontal geometry and normalized to the intensity of features γ and δ. The inset shows a close-up of the spectra near the Fermi level, normalized to feature α, together with the Au Fermi edge (gray solid line). (b) Ti 2*p* core level spectra of TiO*_x_* (*x* = 0.93, 1.06, 1.1, 1.28), and of the Ti^3+^ reference compound YTiO_3_ (dotted line, measured as described in ref 44). The zero of the relative binding energy scale corresponds to 454 eV.

To investigate this issue further, we measured the Ti 2*p* core level spectra, see Figure 1b. We observe one single, asymmetric main peak A_1_/A_2_, which gradually decreases in intensity and shifts slightly to higher binding energies with *x*, and a peak-like protrusion (B) around 3–4 eV above the main peak for *x* = 1.28. In particular, there is no double peak structure with 2 eV separation that resembles the α and β peaks. This is in contrast to cases like V^3+^ and V^4+^ oxides where a close relationship between the lower energy coherent peak in the valence band and a lower energy shake-down feature in the V 2*p* core level spectra was observed.^46,51–53^ We thus can rule out the interpretation that the α and β peaks are the coherent quasiparticle and incoherent lower Hubbard bands, respectively, and we have to find a different origin for this two peak structure in the valence band.

In Figure 1 we also plot the Ti 2*p* core level spectrum of YTiO_3_ (dotted black line) which serves as a Ti^3+^ reference system. We thus can assign feature B to Ti^3+^ 3*d* ^1^ species in TiO_*x*_ with *x* = 1.28. In fact, the 3–4 eV separation between peak B and A_1_/A_2_ fits well with the expected magnitude for the Hubbard *U* in Ti oxides,^54^ so that we can consistently attribute peak A_1_/A_2_ to originate from Ti^2+^ 3*d* ^2^ entities in TiO_*x*_. Since there is no other peak at 3–4 eV lower binding energies, we can rule out the presence of Ti^1+^ 3*d* ^3^ ions even for TiO_*x*_ with *x* < 1. The decrease of peak A_1_/A_2_ with *x* reflects the overall increase of the Ti valence toward less +2 and more +3, and the gradual change in the peak shape from A_1_ to A_2_ requires an explanation involving screening by other than 3*d* electrons. We will show below that these are the Ti 4*s* electrons associated with the presence of oxygen vacancies.

We now return to the valence band and we show the polarization dependence of the HAXPES on TiO_0.93_ in Figure 2a. As we have mentioned earlier, this method allows for the disentanglement of the various atomic subshell contributions to the valence band.^38–41^ Compared to the experiments in the horizontal geometry, the vertical geometry results show a strong suppression of the δ peak at 9 eV and also of the β peak. The α peak, on the other hand, is relatively unaffected. This clearly indicates a very different orbital character of the peaks. Such strong suppression is expected only for *s* orbital contributions,^39–41,55,56^ indicating that peaks δ and β have substantial Ti 4*s* character, while peak α is Ti 3*d* like.

**Figure 2:**
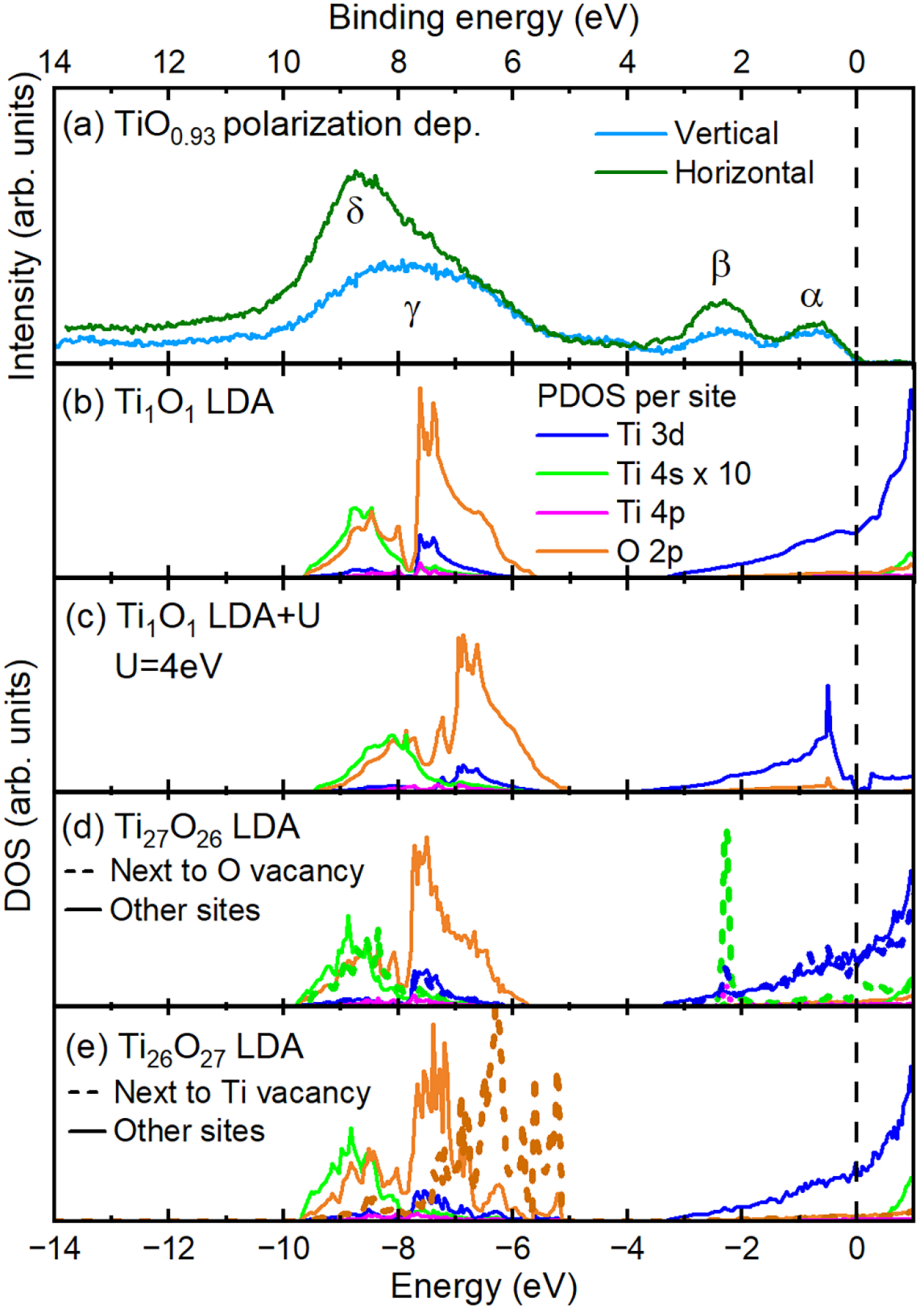
(a) HAXPES valence band spectra of the *x* = 0.93, measured using horizontal and vertical geometries. (b) LDA calculations of rocksalt TiO. The Ti 4*s* pDOS is shown with 10 times multiplication factor (all panels). (c) LDA + *U* calculations of rocksalt TiO, with *U* = 4 eV. (d–e) LDA calculations of an expanded TiO rocksalt cell (3 cells to each direction), with (d) a single O site removed (e) a single Ti site removed. The dashed lines indicate the pDOS at the sites next to the vacancy site, while the solid lines correspond to the average of all remaining sites.

We start with carrying out LDA band structure calculations for the rocksalt Ti_1_O_1_. The results are shown in Figure 2b. We observe that the O 2*p* and Ti 3*d* partial density of states (pDOS) from −9.6 to −5.6 eV can explain well the γ feature of the experimental HAXPES valence band spectrum. Also, as expected from the polarization dependence, the δ structure is well described by the Ti 4*s* pDOS from −9.6 to −7.0 eV, which arises from the hybridization of the TM 4*s* states with the O 2*p* as very commonly found in 3*d* TMOs.^40,41,57–59^ Here we note that we show the Ti 4*s* pDOS multiplied by a factor of 10 in the figure, in order to reflect crudely the large Ti 4*s* photoionization cross-section relative to the Ti 3*d* and O 2*p* in HAXPES.^40,41,55,56^ We will discuss this aspect more quantitatively later. The O 2*p*, Ti 3*d* and Ti 4*s* pDOS from −3.4 to the Fermi level, however, all fail to reproduce peak β as well as the vanishing intensity near the Fermi level in the experiment. For completeness, we further note that the Ti 4*p* states do not contribute much and thus can be safely ignored in the discussions.

While the LDA calculations produce a clear Fermi edge and thus fail to explain the experimentally observed vanishing spectral intensity near the Fermi level, we performed also LDA + *U* calculations with *U* = 4 eV, a very typical value in titanium oxides.^54^ Here we include the spin–orbit interaction in the Ti 3*d* to lift the t_2*g*_ degeneracy. The result is shown in Figure 2c. We are now able to open a band gap, reproducing very well the experiment. This suggests that stoichiometric and defect free TiO should be a *d* ^2^ MH insulator. Yet, these calculations also fail to produce spectral intensity that can be associated with peak β.

Next, to resolve the origin of peak β, we calculate the band structure of oxygen defective TiO_*x*_. Here we took Ti_27_O_26_, i.e., an expanded rocksalt 3 × 3 × 3 supercell with a single O vacancy. The results are displayed in Figure 2d. Compared to the vacancy-free Ti_1_O_1_ case, the changes are negligible, except for the pDOS at the Ti sites next to the O vacancy. There the Ti 4*s* pDOS in the energy region of −3.4 eV upward is completely reconstructed as to form a sharp bound-state-like peak at −2.3 eV. The position of this peak matches very well with that of peak β. We thus have here the spectroscopic signature for the formation of a Ti 4*s* molecular orbital made up by the six Ti ions coordinating each O vacancy. Perhaps one could draw similarities with the formation of donor states around the O vacancies in Eu-rich EuO.^60–62^ In particular, the absence of an O 2*p* pDOS at −2.3 eV shows that this state does not originate from the hybridization of Ti 4*s* with the O 2*p* bands as it is the case for feature δ. The sharpness of the peak also contrasts with the extremely broad itinerant 4*s* bands spanning over several eV as commonly found in the metallic, elemental 3*d* TMs,^55^ indicating its different formation mechanism. We would like to point out that the calculated Ti 3*d* pDOS now also shows an added narrow peak structure at −2.3 eV due to hybridization with the Ti 4*s*, but this effect is small, and in fact so small that it was not followed up further in the literature when it was reported.^28^

While in the Ti_27_O_26_ calculations the 4*s* molecular orbital shows up as a narrow peak, the width of the experimental β feature is larger, of order 1 eV. This suggests that the 4*s* molecular orbital in the real material has sufficient overlap perhaps to even form bands. Indeed, these molecular orbitals involving six Ti ions surrounding an oxygen vacancy are large in spatial extent and will have quickly appreciable overlap for the vacancy concentrations considered.

We have also calculated the band structure of Ti_26_O_27_, i.e., the expanded rocksalt 3 × 3 × 3 supercell with a single Ti vacancy. Figure 2e showed that also here the changes are negligible with respect to the vacancy-free case, except for the pDOS at the O sites next to the Ti vacancy. These changes, however, occur mainly between −8 and −5 eV, i.e., mostly in the vicinity of the top of the O 2*p* band. The more important near Fermi-level region between −3.4 eV and upward remains unaffected.

The band structure calculations provide a natural explanation to the spectral changes observed in TiO_*x*_ for different *x*: Figure 3a shows for the Ti_27_O_26_ solid the O 2*p*, Ti 3*d*, and Ti 4*s* pDOS’ses multiplied by the Fermi function, their respective photoionization cross sections^41,56,65^ for the HAXPES experiment in horizontal geometry, and applying a uniform Gaussian broadening of 450 meV full width at half-maximum to simulate the experimental resolution and lifetime broadening effects. The scale of the broadening required compared to the experimentally determined resolution is on the same order as other valence band HAXPES experiments on transition metal oxides.^41,44^ We observe that the result provides an overall good explanation of the features and intensities in the experimental spectra. In this Ti_27_O_26_ solid we have 6 × 1/27 = 22% of Ti 4*s* on sites next to the O vacancy, and 78% of Ti 4*s* on sites elsewhere. The total sum of these cross-section-weighted pDOS’ses would construct the spectrum for a TiO_*x*_ with 1/27 = 3.7% oxygen vacancies. Figure 3a compares the experimental spectrum of TiO_0.93_ with the calculations in which the amount of Ti 4*s* on sites next to the O vacancy is increased to 44% to simulate a 7% O deficiency. The amount for the other pDOS’s are adjusted accordingly. We can observe that these calculations reproduce the experiment very well, not only in the intensity ratio of the features between −10 to −5 eV on one hand and −3.4 to 0 eV on the other hand, but also in the overall line shape. Similarly, we obtain a good match with the TiO_1.28_ experimental spectrum by using 11% of Ti 4*s* next to the O vacancy, corresponding still to a 1.8% O vacancy content despite the presence of substantial amounts of Ti vacancies.

**Figure 3:**
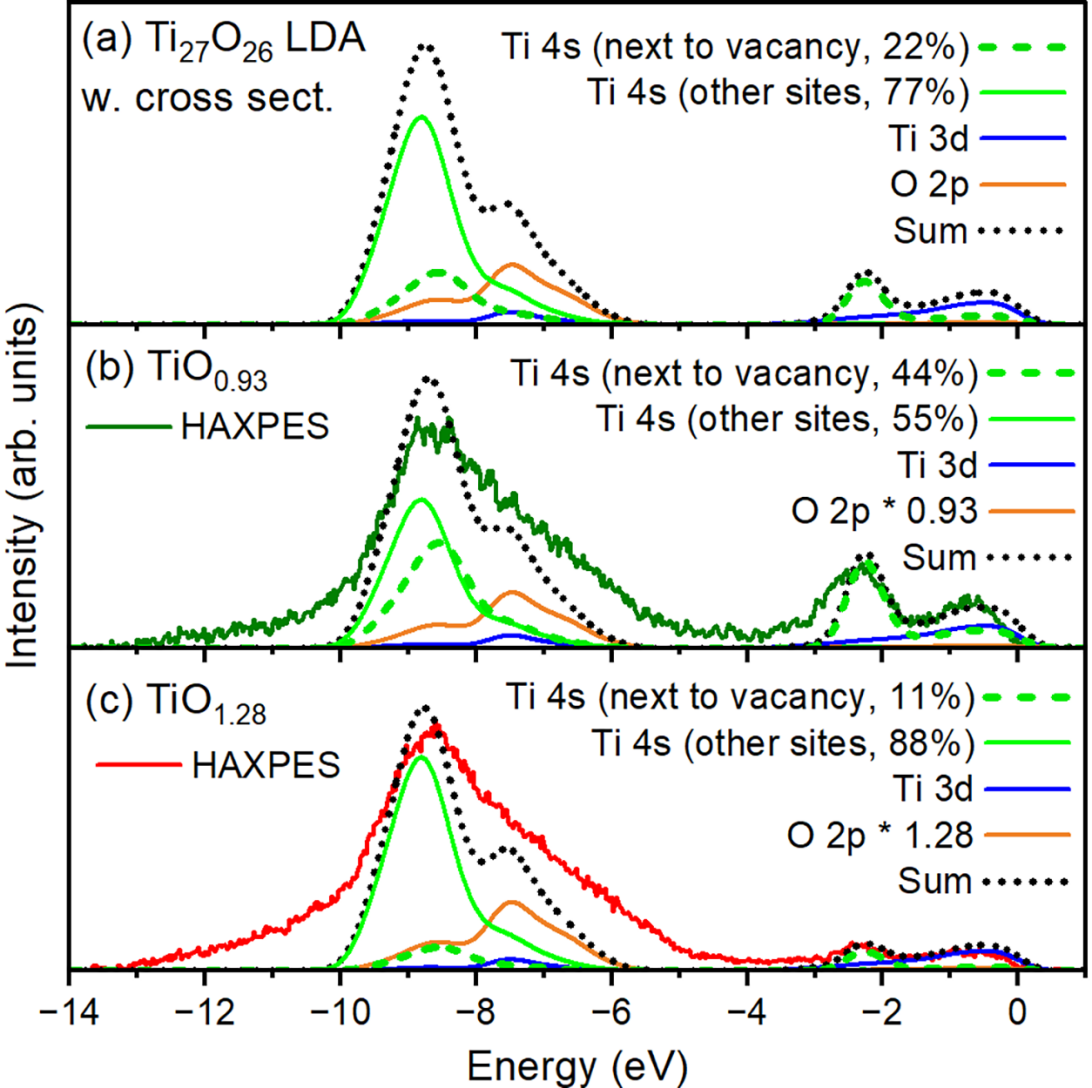
(a) pDOS from the Ti_27_O_26_ multiplied by the photoionization cross sections, with a Gaussian broadening and multiplied by the Fermi function to simulate experimental conditions. (b) and (c) pDOS with a higher and lower amounts of Ti 4*s* on sites next to the O vacancy, to simulate the experimental spectra of TiO_0.93_ and TiO_1.28_, respectively. A Shirley-type integral background^63,64^ has been calculated and subtracted from the experimental spectra to remove the contribution of inelastic processes. The average intensities at the ranges of 0.5–1 eV above and 13–14 eV below the Fermi level have been used as baselines.

### VO*_x_* Results

Figure 4a shows the VO_*x*_ HAXPES valence band spectra. We observe that the features are analogous to those in TiO_*x*_. The spectra display a similarly vanishing intensity at the Fermi level, and also feature β is present which is now at a slightly higher binding energy of around 3 eV. Perhaps in contrast to TiO_*x*_, this β feature disappears in VO_*x*_ for *x* > 1.0. The results obtained with LDA band structure calculations (Figure 4b) are also very similar to those of TiO, with mainly the Fermi level being shifted due to the extra electron per unit cell associated with V replacing Ti. By including a *U* of around 3 eV, we obtain an AFM type I solution with a density of states that matches the experimental spectrum quite well (except for feature β), see Figure 4c. Here the calculations produce a MH gap across the Fermi level and reproduce well the energy positions of features α, γ, and δ. Similar to the TiO_*x*_ case, feature β is not captured by the calculations. The behavior of the V 2*p* core level spectrum, shown in Figure 4d is also similar to that of TiO_*x*_, with the A_1_ asymmetric main peak component gradually decreasing with *x*, and the broad peak B arising around 3–4 eV above the main peak for higher *x* values.

**Figure 4:**
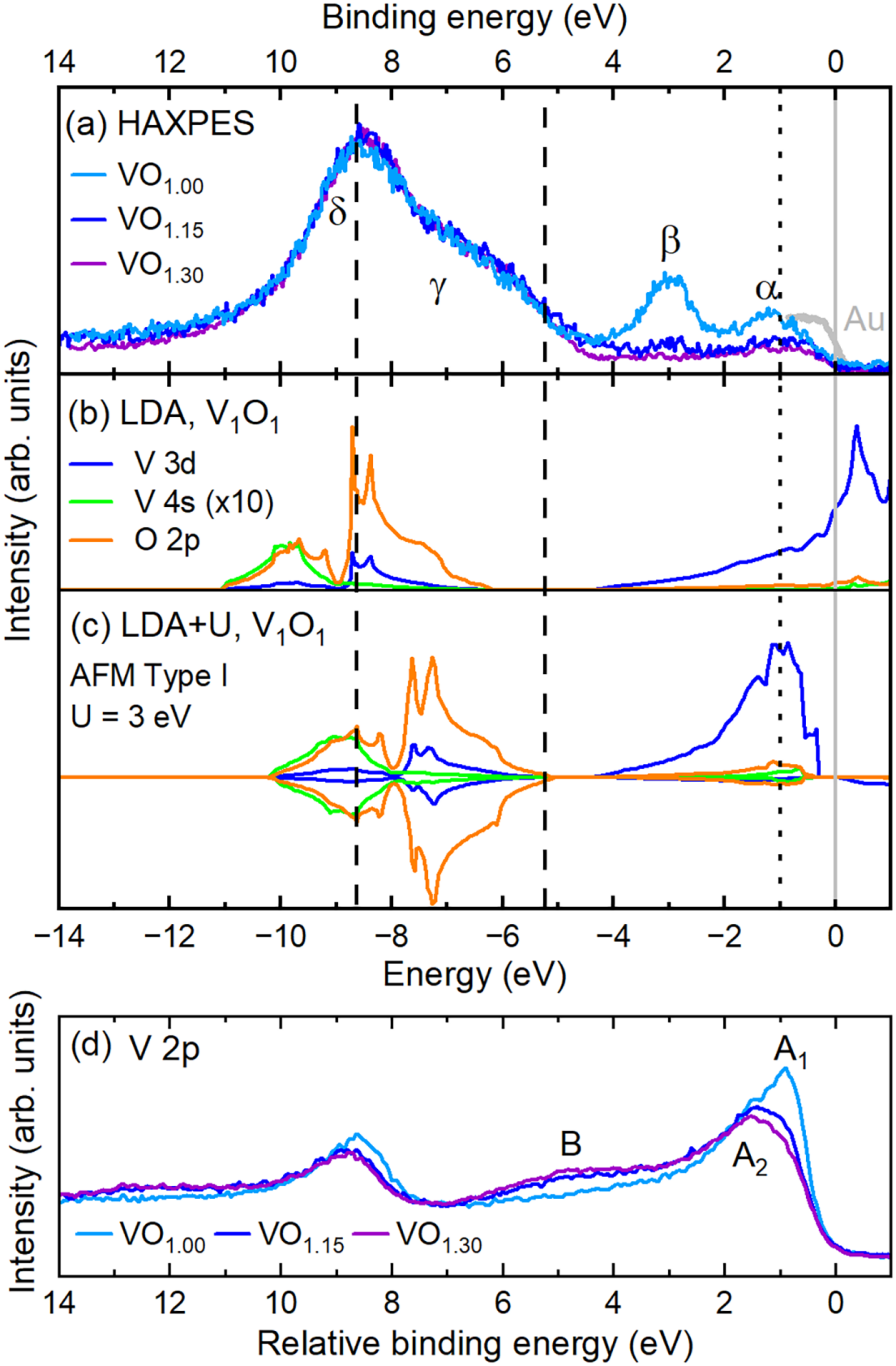
(a) HAXPES valence band spectra of VO*_x_* (*x* = 1.00, 1.15, 1.30), taken with the horizontal geometry and normalized to the intensity of features γ and δ, together with the Au Fermi edge (gray solid line). (b) LDA calculations of rocksalt VO. The V 4*s* pDOS is shown with 10 times multiplication factor. (c) LDA + *U* calculations of rocksalt VO, in an antiferromagnetic type I solution. (d) V 2*p* core level spectra of VO*_x_* (*x* = 1.00, 1.15, 1.30). The zero of the relative binding energy scale corresponds to 512 eV.

Next, we take a closer look at feature β near the Fermi level as displayed in Figure 5, with the spectra now normalized to feature α. Figure 5b shows the V_27_O_26_ LDA calculations to model the band structure of oxygen defective VO_*x*_. Analogous to the TiO_*x*_ case, these calculations show a 4*s* state bound to the sites surrounding the vacancy, confirming thus the presence of similar vacancy states in VO_*x*_ (for *x* ≈ 1). It is worth noting that the 4*s* vacancy states show up here at around 3 eV, fully consistent with the experiment. This consistency for both the TiO_*x*_ and VO_*x*_ strongly supports the concept of 4*s* molecular orbital formation around oxygen vacancies. The observation that the intensity of the β feature quickly diminishes for *x* > 1.0 in VO_*x*_, suggests that oxygen vacancies are no longer present in oxygen-rich VO_*x*_ in contrast to oxygen-rich TiO_*x*_

**Figure 5:**
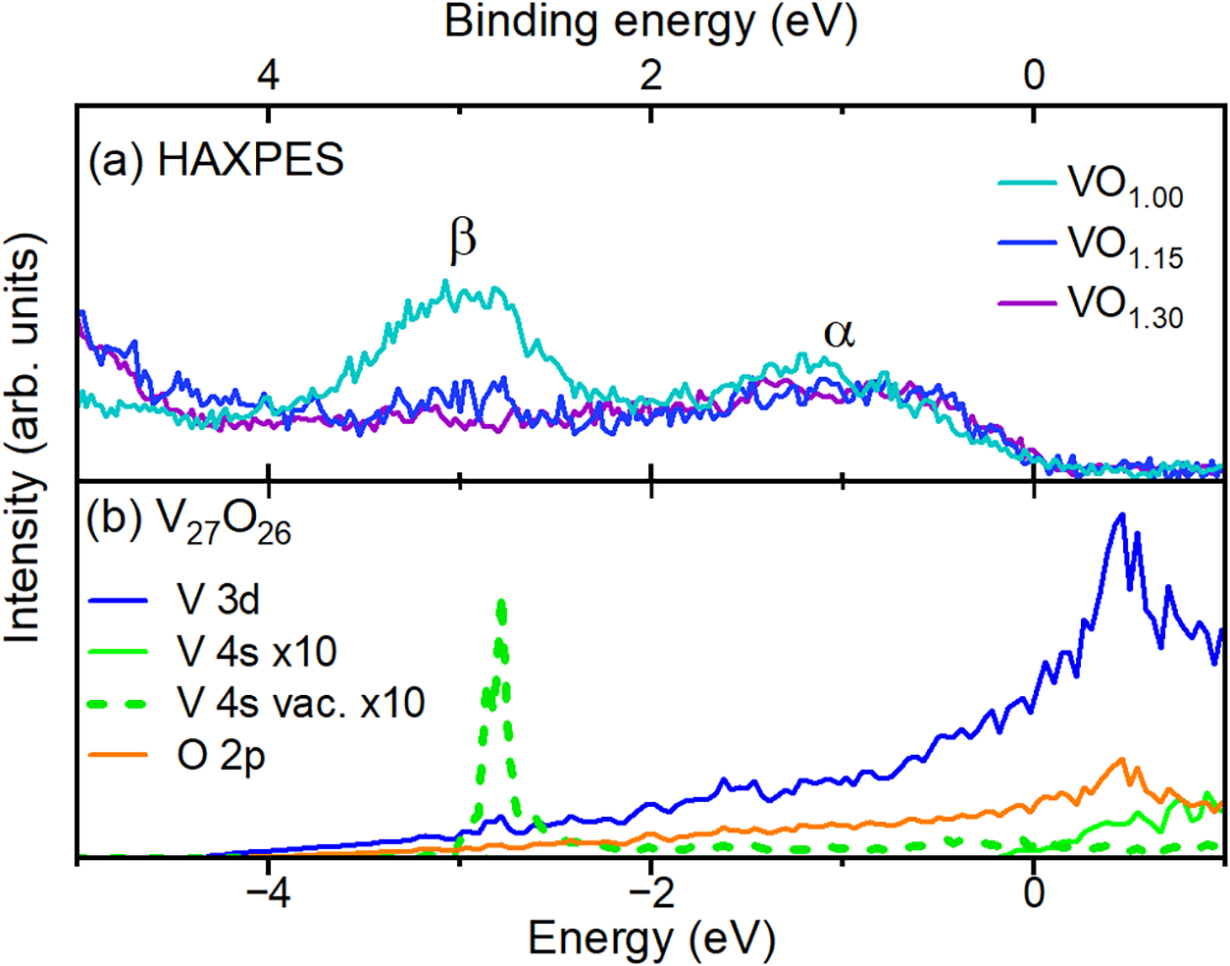
(a) Close-up of the VO*_x_* spectra near the Fermi level, normalized to feature α. (b) LDA calculations of V_27_O_26_ expanded rocksalt cell a single O site removed.

## Discussion

The ability to unveil electronic states of *s* character is essential for the identification of feature β as TM 4*s* molecular orbitals formed around oxygen vacancies in TiO_*x*_ and VO_*x*_. Polarization dependent HAXPES^39–41^ is in this respect one of the most direct and powerful techniques. The information that we can extract from the presence or absence of feature β can now be used to trace back the origin of the conductive properties of these materials. TiO_*x*_ is conductive in its whole composition range while VO_*x*_ only shows a semimetallic behavior for *x* ≲ 1 and becomes more insulating and localized for *x* > 1.^9,10,12,20,21^ At the same time, we observe that feature β is present in the valence band of TiO_*x*_ for all *x* values investigated, while it is only present for *x* ≈ 1 in VO_*x*_. This strongly suggest that the conductivity can be closely linked to the presence of the 4*s* molecular orbitals around the oxygen vacancies. These molecular orbitals are large in spatial extent and will have sufficient overlap for the vacancy concentrations considered. These findings amplify the significance of the 4*s* as an emergent degree of freedom in low-valent TM compounds. Furthermore, from the perspective of the electronic structure, the more insulating behavior in VO_*x*_ for *x* > 1 might then be linked to the fact that the $t_{2g}^{3}$ configuration of V^2+^ favors the octahedral coordination more than the $t_{2g}^{2}$ configuration of Ti^2+^ potentially resulting in O vacancy states around the V site being less stable that those around the Ti site.

Having so far focused on the oxygen vacancies, we now pay our attention to the possible role of the TM vacancies. Here we will make use of our finding that both TiO and VO are in essence so strongly correlated that both would be a MH insulator if the crystals were perfect and stoichiometric. We have seen from Figure 1b signatures for the presence of Ti^3+^ 3*d* ^1^ entities in a background of Ti^2+^ 3*d* ^2^ in TiO_*x*_ with *x* = 1.28. Although in a doped MH insulator the propagation of charges is no longer hampered by the Hubbard *U*, the lattice does react on the different charge states. Indeed, the cation–anion bond distance for a Ti^2+^ ion of about 2.09 Å ^9,43^ is very much larger than for a Ti^3+^ with about 2.03 Å.^66^ This will affect the local Ti energetics considerably, e.g., the hopping integrals with the oxygens can change significantly.^38^ Small polarons can then be formed making the material to become a bad metal, similar like in Fe_3_O_4_ at high temperatures.^67–70^

Yet, with the concentration of oxygen and TM vacancies being very high, substantial overlap between the charge clouds may occur such that vacancy induced bands can be formed. In this respect it is interesting to investigate how the TM–TM, O–O, and TM–O vacancies interact, and to what extent clustering of vacancies can occur. From the experimental side, the pressure-driven control of the vacancies^71,72^ for various x values could give an opportunity to disentangle and explore the effect of TM–TM, O–O and TM–O interactions. On the theoretical side, the presence of electron–lattice interaction and long-range electron–electron interactions have to be considered, as well as the influence of disorder of the vacancies. In this very complex problem, we would like to point out that the MH nature of TiO and VO should also be explicitly included since one may arrive at completely different solutions with regards to energetics, structural relaxations, and stability, than on the basis of uncorrelated band structure studies reported so far.

## Conclusion

In summary, we used bulk-sensitive hard X-ray photoelectron spectroscopy to examine the electronic structure of TiO_*x*_ as well as in VO_*x*_ for a wide range of *x* values. The presence and coexistence of the transition metal (TM) and O vacancies can be identified spectroscopically. We infer from the vanishing spectral intensity at the Fermi level that stoichiometric and defect free rocksalt TiO (VO) are TM^2+^ 3*d* ^2^ (3*d* ^3^) Mott–Hubbard insulators, and that both the TM and O vacancies create localized bound electronic states. The localization associated with TM vacancies is attributed to the formation of small polarons around the TM^3+^ ions. The different behavior with polarization in HAXPES experimentally demonstrates the distinct orbital characters of α and β, with the strong suppression at the vertical geometry indicating significant TM 4*s* weight in β. Band structure calculations provide a very direct connection between the 4*s*-dominant β feature and the well-known presence of oxygen vacancies in TiO_*x*_ and VO_*x*_ i.e., molecular orbitals of TM 4*s* states forming at the TM ions surrounding the oxygen vacancy. Furthermore, the vanishing of β for VO_*x*_, *x* > 1 where the system becomes more insulating suggests a direct relation between the presence of these molecular orbitals and the conducting properties. Our study enables further in-depth theoretical and experimental studies with the new *Ansatz*, aimed at exploring the role of these molecular orbital states in TiO_*x*_ and VO_*x*_.

In TMO’s, the 4*s* states are considered in general to be rather passive with their role being limited to hybridization with the O 2*p* contributing only to the chemical bonding. The unexpected discovery of the presence of these 4*s* molecular orbitals suggests the importance of TM 4*s* orbitals as an emerging degree of freedom providing new possibilities in low-valent transition-metal oxides, and opens up a new path for the realization of the physical and chemical control of the vacancy-driven 4s states in these compounds.

## Supplementary Material



## Notes

LDA + *U* calculations performed on the expanded TiO rocksalt cell with a single O site removed, and supplementary Figure S1 comparing the calculations with the experimental spectra and the LDA (PDF)
