# Supplementary material for: 4s Molecular Orbitals and Strongly Correlated 3d States in TiO x and VO x
Source: J Am Chem Soc. 2026 Apr 17;148(16):16821–9. doi: 10.1021/jacs.5c22806 (PMC13134633; doi:10.1021/jacs.5c22806)
Supplement: Supplementary file 1 [file ja5c22806_si_001.pdf]

Supporting Information for "4s Molecular Orbitals and Strongly Correlated 3d States in TiO_x and VO_x"

Daisuke Takegami*[1,2,3], Anna Melendez-Sans[2], Takashi Miyoshino[1], Ryo Nakamura[1], Miguel Ferreira-Carvalho[4,2], Georg Poelchen[2], Chun-Fu Chang[2], Masato Yoshimura[5], Ku-Ding Tsuei[5], Haruka Matsumoto[6], Asuka Yanagida[6], Ryota Yoshimura[6], Suguru Yano[6], Takumi Iwata[6], Takuro Katsufuji[6], Atsushi Hariki[7], Liu Hao Tjeng[2], and Takashi Mizokawa[1]

[1]Department of Applied Physics, Waseda University, Shinjuku, Tokyo 169-8555, Japan
[2]Max Planck Institute for Chemical Physics of Solids, Nöthnitzer Straße 40, Dresden 01187, Germany
[3]Department of Physics, Tokyo Metropolitan University, Hachioji 192-0397, Japan
[4]Institute of Physics II, University of Cologne, Zülpicher Str. 77, Cologne D-50937, Germany
[5]National Synchrotron Radiation Research Center, Hsinchu 30076, Taiwan
[6]Department of Physics, Waseda University, Shinjuku, Tokyo 169-8555, Japan
[7]Department of Physics and Electronics, Graduate School of Engineering, Osaka Metropolitan University, 1-1 Gakuen-cho, Nakaku, Sakai, Osaka 599-8531, Japan

*Email: dtakegami@tmu.ac.jp

Additional calculations

LDA+U (U=4 eV) calculations on the expanded TiO rocksalt cell with a single O site removed have also been performed for a reduced k-mesh of $6 \times 6 \times 6$, with the results displayed on Fig. S1(b), together with the experimental data (a) and the calculations without U (c) shown in the main paper. We observe that the results present both the suppression at the Fermi level that was shown for the LDA+U calculations on the ideal rocksalt structure, as well as the Ti 4s-derived β feature. Here, the peak β is split into two separate components due to the exchange interactions. The peak positions, as well as the broadening due to are still equally compatible with the experimental spectra.

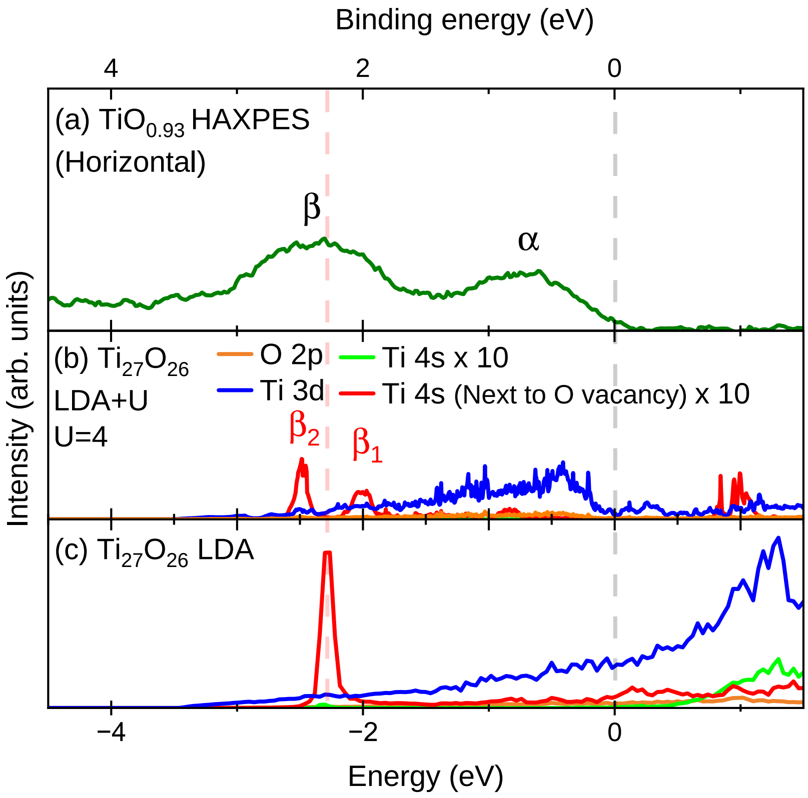

Figure S1: (a) HAXPES valence band spectra of the x=0.93 measured using the horizontal geometry. (b) LDA+U (U=4 eV) calculations of the expanded TiO rocksalt cell with a single O site removed. The Ti 4s pDOS is shown with 10 times multiplication factor (all panels). (c) LDA calculations of the expanded TiO rocksalt cell with a single O site removed.
